# Causal Estimands for Analyses of Averted and Avertible Outcomes due to Infectious Disease Interventions

**DOI:** 10.1097/EDE.0000000000001839

**Published:** 2025-01-24

**Authors:** Katherine M. Jia, Christopher B. Boyer, Jacco Wallinga, Marc Lipsitch

**Affiliations:** From the ^a^Center for Communicable Disease Dynamics, Department of Epidemiology, Harvard T.H. Chan School of Public Health, Boston, MA; b Centre for Infectious Disease Control, National Institute for Public Health and the Environment, Bilthoven, The Netherlands; c Department of Biomedical Data Sciences, Leiden University Medical Center, Leiden, The Netherlands; d Department of Immunology and Infectious Diseases, Harvard T.H. Chan School of Public Health, Boston, MA.

**Keywords:** Direct effect, Overall effect, Vaccine-averted deaths, Vaccine-preventable deaths

## Abstract

During the coronavirus disease (COVID-19) pandemic, researchers attempted to estimate the number of averted and avertible outcomes due to vaccination campaigns to quantify public health impact. However, the estimands used in these analyses have not been previously formalized. It is also unclear how these analyses relate to the broader framework of direct, indirect, total, and overall causal effects under interference. Here, using potential outcome notation, we adjust the direct and overall effects to accommodate analyses of averted and avertible outcomes. We use this framework to interrogate the commonly held assumption that vaccine-averted outcomes via direct impact among vaccinated individuals (or vaccine-avertible outcomes via direct impact among unvaccinated individuals) is a lower bound on vaccine-averted (or -avertible) outcomes overall. To do so, we describe a susceptible-infected-recovered-death model stratified by vaccination status. When vaccine efficacies wane, the lower bound fails for vaccine-avertible outcomes. When transmission or fatality parameters increase over time, the lower bound fails for both vaccine-averted and -avertible outcomes. Only in the simplest scenario where vaccine efficacies, transmission, and fatality parameters are constant over time, outcomes averted via direct impact among vaccinated individuals (or outcomes avertible via direct impact among unvaccinated individuals) is a lower bound on overall impact. In conclusion, the lower bound can fail under common violations to assumptions on time-invariant vaccine efficacy, pathogen properties, or behavioral parameters. In real data analyses, estimating what seems like a lower bound on overall impact through estimating direct impact may be inadvisable without examining the directions of indirect effects.

Recently, researchers have estimated the number of COVID-19 deaths (or infections) averted by vaccination campaigns in the United States,^1,2^ Israel,^3,4^ Chile,^5^ Brazil,^6^ and Japan.^7^ Similarly, other studies, including our own,^8^ have attempted to estimate vaccine-avertible deaths—defined as the number of deaths that could have been averted by vaccination but were not because of a failure to vaccinate the unvaccinated.^8,9^ Most empirical studies quantify direct protection conferred by vaccination among vaccinated individuals, but they typically assume the overall impact of vaccination is larger due to indirect protection.^4–6^ Indeed, these studies often claim that their estimates represent a lower bound on the overall impact of vaccination, although this claim has not been carefully verified.

To support this claim, many of these studies draw, either implicitly or explicitly, on the causal effect framework developed by Halloran and Struchiner,^10^ in which the overall effect (OE) of vaccines can be decomposed into direct and indirect components.^11^ However, the Halloran and Struchiner framework has not yet been formally extended to cover the specific estimands targeted in vaccine-averted and -avertable analyses, which estimate impact. Existing effect estimands are defined by contrasts of individual risk^12,13^ and have been used to estimate vaccine efficacy (VE) in clinical trials.^14,15^ However, observational studies, postlicensure studies, and policymakers are often equally or more interested in quantifying the public health impact of vaccination in terms of counts^16^ such as the number of infections, hospitalizations, and deaths in a group of individuals, instead of risk in each individual.

Motivated by recent empirical studies on vaccine-averted and -avertible COVID-19 deaths, this article seeks to fill these gaps by: (1) clearly defining impact estimands as corollaries of direct and overall effect estimands for averted and avertible outcomes (i.e., counts), and (2) determining the conditions under which direct impact is a lower bound on overall impact. To ground our discussion, we introduce a susceptible-infected-recovered-death model stratified by vaccination status to investigate direct and overall impact of vaccination under different scenarios.

The remainder of this article is organized as follows. We first summarize the definitions of direct effect (DE), indirect effect (IE), total effect (TE), and OE introduced by Hudgens and Halloran,^12^ and provide an alternative partitioning of OE into components that will align better with the estimands targeted by vaccine-averted and avertible analyses. Next, we propose count outcome corollaries for direct and overall effects, show how they map onto estimands for vaccine-averted and -avertible analyses, and formalize the claim that direct impact constitutes a lower bound on overall impact. Then, we outline a transmission model to simulate vaccine-averted and -avertible outcomes. Finally, we examine the conditions under which outcomes averted via direct impact among vaccinated individuals (or outcomes avertible via direct impact among unvaccinated individuals) is or is not a lower bound on the overall impact on vaccine-averted (or -avertible) outcomes.

## DIRECT, INDIRECT, OVERALL, AND TOTAL EFFECTS

Hudgens and Halloran^12^ previously defined causal estimands for DE, IE, OE, and TE in the two-stage randomized trial, as summarized in the following section.

### Setup and Notation

Consider a two-stage randomized trial with m groups indexed by i=1,…,m, such that each group consists of N individuals indexed by j=1,…,N with a large group size N. All groups are assumed to be of same size N. Partial interference is assumed: individuals make contacts within the same group, but individuals in different groups make no contacts. For ease of exposition, assume interest lies in quantifying the effect of vaccination, which is a one-time event before the start of an outbreak. Let $A_{ij}=1$ if individual j in each group i is vaccinated and $A_{ij}=0$ otherwise. Let $A_{i}=\left(A_{i1},A_{i2},\ldots ,A_{iN}\right)$ and $A_{i,-j}=\left(A_{i1},A_{i2},\ldots ,A_{ij-1},A_{ij+1},\ldots ,A_{iN}\right)$, hereafter referred to as allocation programs.^11^ Let $a_{i}$ and $a_{i,-j}$ denote possible realizations of $A_{i}$ and $A_{i,-j}$, respectively. Let A(N) denote the set of all possible $2^{N}$ vaccine allocations for a group of size N, for which $a_{i}\in A\left(N\right)$.

Let $Y_{ij}\left(a_{i}\right)$ denote the potential binary outcome for individual j in group i with allocation program $a_{i}$ and let $Y_{ij}\left(a_{i,-j},a\right)$ denote the potential binary outcome when individual j has vaccination status a and the rest of group i has vaccination status $a_{i,-j}$.

### Individual, Group, and Population Average Potential Outcomes

Hudgens and Halloran^12^ define marginal individual average potential outcome as


$$\begin{array}{l} {\bar{Y}}_{ij}(\alpha )\equiv \sum_{s\in A(N)}Y_{ij}(a_{i}=s)\underset{\alpha }{\mathrm{Pr}}(A_{i}=s) \end{array}$$

and individual average potential outcome^12^ as:


$$\begin{array}{l} {\bar{Y}}_{ij}(a;\alpha )\equiv \sum_{s\in A(N-1)}Y_{ij}(a_{i,-j}=s,a_{ij}=a)\underset{\alpha }{\mathrm{Pr}}(A_{i,-j}=s|A_{ij}=a) \end{array}$$

where $\underset{\alpha }{\mathrm{Pr}}\left(\cdot \right)$ is the probability distribution of vaccine allocation program $A_{i}$ with parameter α∈[0,1] representing the proportion vaccinated within group i. Specifically, $\underset{\alpha }{\mathrm{Pr}}\left(\cdot \right)$ is the probability distribution of $A_{i}$ conditional on $\underset{j}{\overset{N}{\sum }}\;A_{ij}=\alpha \cdot N$. Note here we use type A parameterization, which gives same effect definitions as type B parameterization suggested by VanderWeele and Tchetgen Tchetgen^11^ when N is large. Definitions of $\underset{\alpha }{\mathrm{Pr}}\left(\cdot \right)$, type A, and type B parameterizations are given in eAppendix 1; http://links.lww.com/EDE/C222. Hudgens and Halloran^12^ further define (marginal) group average potential outcomes ${\bar{Y}}_{i}\left(\alpha \right)\equiv \overset{N}{\underset{j=1}{\sum }}{\bar{Y}}_{ij}\left(\alpha \right)/N$ and ${\bar{Y}}_{i}\left(a;\alpha \right)\equiv \overset{N}{\underset{j=1}{\sum }}{\bar{Y}}_{ij}\left(\;a;\alpha \right)/N$ by averaging over individuals within groups. They also define (marginal) population average potential outcomes^12^ $\bar{Y}\left(\alpha \right)=\overset{m}{\underset{i=1}{\sum }}{\bar{Y}}_{i}\left(\alpha \right)/m$ and $\bar{Y}\left(a;\alpha \right)=\overset{m}{\underset{i=1}{\sum }}{\bar{Y}}_{i}\left(a;\alpha \right)/m$.

### Population Average Direct, Indirect, Overall, and Total Causal Effects

Hudgens and Halloran define population average direct casual effect^12^ as $DE\left(\alpha \right)=\bar{Y}\left(0;\alpha \right)-\bar{Y}\left(1;\alpha \right)$, comparing $\bar{Y}$ when an individual is unvaccinated versus when vaccinated, holding fixed proportion vaccinated (α). They define population average indirect casual effect^12^ as $IE^{Unvax}\left(\alpha ,\alpha^{\prime }\right)=\bar{Y}\left(0;\alpha \right)-\bar{Y}\left(0;\alpha^{\prime }\right)$, comparing $\bar{Y}$ for an unvaccinated individual in a group with α proportion vaccinated versus with $\alpha^{\prime }$, hereafter referred to as IE for the unvaccinated. As suggested previously,^17^ IE can be analogously defined for a vaccinated individual: $IE^{Vax}\left(\alpha ,\alpha^{\prime }\right)=\bar{Y}\left(1;\alpha \right)-\bar{Y}\left(1;\alpha^{\prime }\right)$, hereafter referred to as IE for the vaccinated. They also define population average total causal effect^12^ as $TE\left(\alpha ,\alpha^{\prime }\right)=\bar{Y}\left(0;\alpha \right)-\bar{Y}\left(1;\alpha^{\prime }\right)$, comparing $\bar{Y}$ for an individual when they are unvaccinated in a group with α proportion vaccinated versus when they are vaccinated in a group with $\alpha^{\prime }$. Following from the definition, $TE\left(\alpha ,\alpha^{\prime }\right)$ can be decomposed as $DE\left(\alpha^{\prime }\right)+IE^{Unvax}\left(\alpha ,\alpha^{\prime }\right)$ or $DE\left(\alpha \right)+IE^{Vax}\left(\alpha ,\alpha^{\prime }\right)$. Finally, they define population average overall casual effect^12^ as $OE\left(\alpha ,\alpha^{\prime }\right)=\bar{Y}\left(\alpha \right)-\bar{Y}\left(\alpha^{\prime }\right)$, comparing $\bar{Y}$ for a typical individual in a group with α proportion vaccinated versus with $\alpha^{\prime }$.

### Overall Effect Partitioning

Previously, Hudgens and Halloran^12^ showed that when α=0, $OE\left(\alpha ,\alpha^{\prime }\right)$ is the weighted sum $\alpha^{\prime }\cdot TE\left(\alpha ,\;\alpha^{\prime }\right)+\left(1-\alpha^{\prime }\right)\cdot IE^{Unvax}\left(\alpha ,\alpha^{\prime }\right)$. Here, to establish direct impact as a lower bound on overall impact (see next section), we generalize $OE\left(\alpha ,\alpha^{\prime }\right)$ partitioning to any $\;\;\;\alpha^{\prime }>\alpha$ in Theorem 1.

*Theorem 1 (OE partitioning*)


$$\begin{array}{l} \begin{array}{ll} & OE\left(\alpha ,\alpha^{\prime }\right)=\alpha \cdot IE^{Vax}\left(\alpha ,\alpha^{\prime }\right)+\left(\alpha^{\prime }-\alpha \right)\cdot TE\left(\alpha ,\;\alpha^{\prime }\right)\;\\ & +\left(1-\alpha^{\prime }\right)\cdot IE^{Unvax}\left(\alpha ,\alpha^{\prime }\right).\;\;\; \end{array} \end{array}$$

**FIGURE 1.**
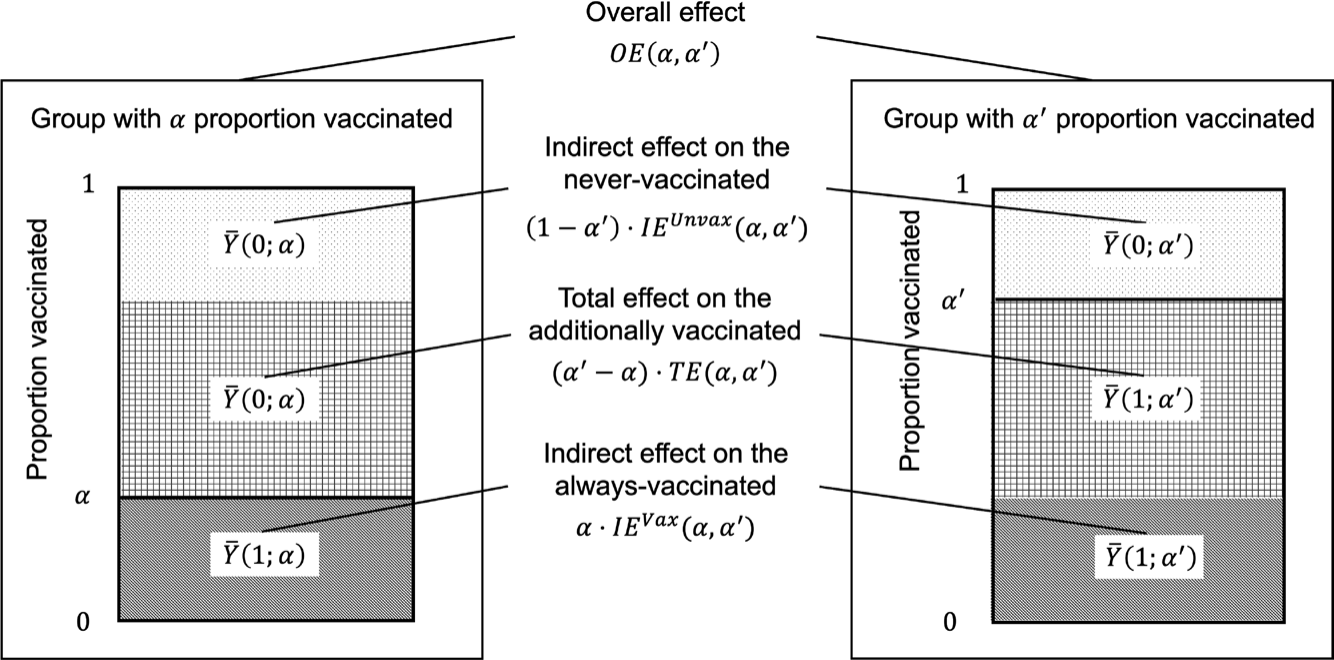
Graphical illustration on partitioning overall effect. The two rectangles represent a pair of counterfactuals wherein the group has α or $\alpha^{\prime }$ proportion vaccinated ($\alpha^{\prime }>\alpha$). Individuals fall into three categories based on their vaccination status under the counterfactuals: (1) The dotted region represents those who are unvaccinated under both counterfactuals (hereafter referred to as “never vaccinated”) and for whom $IE^{Unvax}\left(\alpha ,\alpha^{\prime }\right)$ is in operation; (2) the gridded region represents those who are unvaccinated under α but vaccinated under $\alpha^{\prime }$ and for whom $TE\left(\alpha ,\alpha^{\prime }\right)$ is in operation (hereafter referred to as “additionally vaccinated”); and (3) the stripped region represents those who are vaccinated under both counterfactuals (hereafter referred to as “always vaccinated”) and for whom $IE^{Vax}\left(\alpha ,\alpha^{\prime }\right)$ is in operation. Theorem 1 shows that $OE\left(\alpha ,\alpha^{\prime }\right)$ is a weighted average of three effects: (1) $IE^{Unvax}\left(\alpha ,\alpha^{\prime }\right)$, (2) $TE\left(\alpha ,\alpha^{\prime }\right)$, and (3) $IE^{Vax}\left(\alpha ,\alpha^{\prime }\right)$, each weighted by the proportion of individuals for whom the effect is in operation, respectively: (1) $1-\alpha^{\prime }$ for the never vaccinated, (2) $\alpha^{\prime }-\alpha$ for the additionally vaccinated, and (3) α for the always vaccinated.

Theorem 1 is proven in eAppendix 2; http://links.lww.com/EDE/C222 and graphically illustrated in Figure 1. Theorem 1 expresses $OE\left(\alpha ,\alpha^{\prime }\right)$ as a weighted average of three effects: (1) $IE^{Vax}\left(\alpha ,\alpha^{\prime }\right)$, (2) $TE\left(\alpha ,\alpha^{\prime }\right)$, and (3) $IE^{Unvax}\left(\alpha ,\alpha^{\prime }\right)$. Intuitively, if individuals are classified by vaccination status under a pair of counterfactuals wherein the group has α or $\alpha^{\prime }$ proportion vaccinated ($\alpha^{\prime }>\alpha$), then $IE^{Vax}\left(\alpha ,\alpha^{\prime }\right)$ is in operation for proportion α of individuals who are vaccinated under both counterfactuals; $TE\left(\alpha ,\alpha^{\prime }\right)$ is in operation for proportion $\alpha^{\prime }-\alpha$ of individuals who are vaccinated under $\alpha^{\prime }$ but not α; and $IE^{Unvax}\left(\alpha ,\alpha^{\prime }\right)$ is in operation for proportion $1-\alpha^{\prime }$ of individuals who are unvaccinated under both counterfactuals.

## ESTIMANDS FOR ANALYSES OF AVERTED AND AVERTIBLE OUTCOMES

Up until now, all effect estimands (except IE for vaccinated)^17^ have been previously defined by Hudgens and Halloran in a two-stage randomized trial. To define estimands for analyses of averted and avertible outcomes, we now expand on the terminology of two-stage randomization. Although a two-stage randomized trial is rarely conducted in practice, our goal is to be explicit about the definition of estimands in recent observational studies on averted and avertible outcomes and to place these observational studies in the context of target trial emulation, which is playing a growing role in causal inference.

The original OE and DE are defined for individual risk,^10^ and their magnitudes are not comparable because they use different denominators. However, empirical observational studies of averted and avertible deaths use count outcomes, assuming vaccination prevents more deaths overall than directly (i.e., OE multiplied by total population size is greater than multiplying DE with a number of vaccinated individuals in the presence of indirect protection for unvaccinated individuals).^4–9^ To verify this assumption, in this section, we define two estimands for averted and avertible outcomes—direct impact and overall impact—that align with estimands targeted by literature on vaccine-averted and -avertible outcomes.^1–9^ In the sections that follow, we verify conditions under which direct impact is or is not a lower bound on overall impact.

This article adds to existing literature by: (1) introducing an additional time index t to examine outcomes at multiple time points postvaccination, (2) defining direct and overall impact (i.e., corollaries of direct and overall effect estimands for count outcomes) to be used in averted and avertible outcome analyses, (3) defining the relationships between direct and overall impact, and (4) most importantly, using analytical and simulation approaches to identify conditions under which direct impact may or may not be a lower bound on overall impact.

### Notation

To examine direct and overall impact over time, we incorporate a time index t so that $\bar{Y}\left(t,a;\alpha \right)$ denotes population average cumulative incidence of having developed the outcome by time t, and similarly for $\bar{Y}\left(t;\alpha \right)$ and all the effect estimands in the previous section. t=0 denotes start of follow-up. In principle, t can be of any timescale, while t is in days in the simulations.

Now let $\alpha_{1}$ denote a particular vaccination proportion chosen to be implemented in the group, let $\alpha_{2}$ denote some hypothetical higher proportion (i.e., $\alpha_{2}>\alpha_{1}$), and let $\alpha_{0}$ denote some hypothetical lower proportion (i.e., $\alpha_{0}<\alpha_{1}$).

### Motivating Examples and Causal Questions

During the COVID-19 pandemic, determining the total number of infections (or deaths) averted by vaccination has been of great public health interest.^1–7,18–20^ Vaccine-averted infections (or deaths) is an impact estimand based on the causal question: How many infections (or deaths) have been averted under the particular proportion vaccinated ($\alpha_{1}$) compared to the counterfactual in the absence of vaccination ($\alpha_{0}=0$)?

Alternatively, researchers have also estimated the vaccine-avertible deaths—those that could have been averted by vaccination but were not because of a failure to vaccinate.^8,9^ The causal question is: How many infections (or deaths) could have been averted under full vaccination ($\alpha_{2}=1$), but were not averted given the particular proportion vaccinated ($\alpha_{1}$)?

In general, estimands are defined by comparing number of infections (or deaths) in a typical group under the particular proportion vaccinated ($\alpha_{1}$) versus a lower ($\alpha_{0}$) or higher ($\alpha_{2}$) proportion.^9^ We term these as impact estimands because previous literature has referred to vaccine-averted infections (or deaths) as (population) impact of vaccination.^5,7,18,19^

### Overall Impact

For $\alpha_{0}<\alpha_{1}$, overall impact is defined as:

$$\begin{array}{l} \delta^{O}\left(t,\alpha_{0},\alpha_{1}\right)=N\cdot OE\left(t,\alpha_{0},\alpha_{1}\right). \end{array}$$ (1.1)

For $\alpha_{2}>\alpha_{1}$, overall impact is defined as:

$$\begin{array}{l} \delta^{O}\left(t,\alpha_{1},\alpha_{2}\right)=N\cdot OE\left(t,\alpha_{1},\alpha_{2}\right).\;\;\; \end{array}$$ (1.2)

Overall impact directly answers the two aforementioned causal questions on quantifying vaccine-averted and -avertible outcomes. For reasons clarified later, we force overall impact to have the same sign when $\alpha_{1}$ is compared to a lower ($\alpha_{0}$) or higher ($\alpha_{2}$) hypothetical value.

Note mathematical modeling studies implicitly refer to overall impact when estimating vaccine-averted deaths by simulating the epidemic trajectory under a hypothetical proportion vaccinated (e.g., $\alpha_{0}=0$) and comparing it with the trajectory under the particular vaccination campaign ($\alpha_{1}$).

Furthermore, by Theorem 1 and $TE\left(t,\;\alpha_{0},\;\alpha_{1}\right)=DE\left(t,\alpha_{1}\right)+IE^{Unvax}\left(t,\alpha_{0},\alpha_{1}\right)$, we decompose:

$$\begin{array}{l} \begin{array}{ll} & \delta^{O}\left(t,\alpha_{0},\alpha_{1}\right)=N\cdot OE\left(t,\alpha_{0},\alpha_{1}\right)\;\\ & \;\;\;\;\;\;\;\;\;\;\;\;\;\;\;\;\;\;\;\;\;\;\;\;\;\;=N\cdot \alpha_{0}\cdot IE^{Vax}\left(t,\alpha_{0},\alpha_{1}\right)\;\\ & +\;N\cdot \left(\alpha_{1}-\alpha_{0}\right)\cdot \left(DE\left(t,\alpha_{1}\right)+IE^{Unvax}\left(t,\alpha_{0},\alpha_{1}\right)\right)\;\\ & \;\;\;\;\;\;\;\;\;\;\;\;\;\;\;\;\;\;\;\;\;\;\;\;\;\;\;+\;N\cdot \left(1-\alpha_{1}\right)\cdot IE^{Unvax}\left(t,\alpha_{0},\alpha_{1}\right)\;\\ & \;\;\;\;\;\;\;\;\;\;\;\;\;\;\;\;\;\;\;\;\;\;\;\;\;\;=N\cdot \alpha_{0}\cdot IE^{Vax}\left(t,\alpha_{0},\alpha_{1}\right)\;\\ & +\;N\cdot \left(\alpha_{1}-\alpha_{0}\right)\cdot DE\left(t,\alpha_{1}\right)+N\cdot \left(1-\alpha_{0}\right)\;\\ & \;\;\;\;\;\;\;\;\;\;\;\;\;\;\;\;\;\;\;\;\;\;\;\;\;\;\;\;\cdot \;IE^{Unvax}\left(t,\alpha_{0},\alpha_{1}\right)\; \end{array} \end{array}$$ (2.1)

for $\alpha_{1}>\alpha_{0}$. The first term on the right-hand side of last line of equation (2.1) scales $IE^{Vax}\left(t,\alpha_{0},\alpha_{1}\right)$ by number vaccinated under $\alpha_{0}$. The second term scales $DE\left(t,\alpha_{1}\right)$ by the additional number vaccinated under $\alpha_{1}$ compared to $\alpha_{0}$. The third term scales $IE^{Unvax}\left(t,\alpha_{0},\alpha_{1}\right)$ by number unvaccinated under $\alpha_{0}$.

Similarly, by Theorem 1 and $TE\left(t,\;\alpha_{1},\;\alpha_{2}\right)=DE\left(t,\alpha_{1}\right)+IE^{Vax}\left(t,\alpha_{1},\alpha_{2}\right)$, we decompose:

$$\begin{array}{l} \begin{array}{ll} & \delta^{O}\left(t,\alpha_{1},\alpha_{2}\right)=N\cdot OE\left(t,\alpha_{1},\alpha_{2}\right)\;\\ & =N\cdot \alpha_{2}\cdot IE^{Vax}\left(t,\alpha_{1},\alpha_{2}\right)\;\\ & +N\cdot \left(\alpha_{2}-\alpha_{1}\right)\cdot DE\left(t,\alpha_{1}\right)\;\\ & +N\cdot \left(1-\alpha_{2}\right)\cdot IE^{Unvax}\left(t,\alpha_{1},\alpha_{2}\right)\; \end{array} \end{array}$$ (2.2)

for $\alpha_{2}>\alpha_{1}$. The first term on the right-hand side of last line of equation (2.2) scales $IE^{Vax}\left(t,\alpha_{1},\alpha_{2}\right)$ by number vaccinated under $\alpha_{2}$. The second term scales $DE\left(t,\alpha_{1}\right)$ by the additional number vaccinated under $\alpha_{2}$ compared to $\alpha_{1}$. The third term scales $IE^{Unvax}\left(t,\alpha_{1},\alpha_{2}\right)$ by number unvaccinated under $\alpha_{2}$.

### Direct Impact

Because overall impact ($\delta^{O}$) contrasts two different vaccination proportions, at minimum researchers need to observe two noninteracting groups where each is “assigned” to one of the vaccination strategies to estimate overall impact.^12^ Ideally, vaccination would be assigned via a two-stage randomized trial. However, these trials are rare because they are expensive and hard to justify.^21^ Instead, most empirical studies only observe a single group under one vaccination proportion ($\alpha_{1}$), in which case researchers can only estimate an impact corollary of DE. Examples include observational studies comparing discrete hazards or incidence rates between vaccinated and unvaccinated individuals based on national vaccine data systems.^4–9^ These studies multiply DE with number vaccinated (or unvaccinated) to estimate outcomes averted (or avertible) via direct impact among vaccinated (or unvaccinated) individuals,^4–9^ and then generally assume that it is lower than overall impact in the entire population.^4–6,8^ Here, we formalize this definition of direct impact and show how it relates to overall impact.

Some empirical observational studies have estimated deaths averted via direct impact among vaccinated individuals^4–7^ using formulas similar to $N\cdot \left(\alpha_{1}-\alpha_{0}\right)\cdot DE\left(t,\alpha_{1}\right)$ by setting $\alpha_{0}=0$, while others have estimated deaths avertible via direct impact among unvaccinated individuals^8,9^ using formulas similar to $N\cdot \left(\alpha_{2}-\alpha_{1}\right)\cdot DE\left(t,\alpha_{1}\right)$ by setting $\alpha_{2}>\alpha_{1}$ (note these studies have also considered increases in proportion vaccinated over time, such as under a vaccine rollout, which here we ignore for simplicity). Following the literature,^4–9,18^ let direct impact ($\delta^{D}$) for any α, $\alpha^{\prime }\in \left[0,1\right]$ be $\delta^{D}\left(t,\alpha ,\alpha {\;}^{\prime }\;\right)=N\cdot \left|\alpha^{\prime }-\alpha \right|\cdot DE\left(t,\alpha^{\prime }\right).$

In particular, for $\alpha_{1}>\alpha_{0}$, we have

$$\begin{array}{l} \delta^{D}\left(t,\alpha_{0},\alpha_{1}\right)=N\cdot \left(\alpha_{1}-\alpha_{0}\right)\cdot DE\left(t,\alpha_{1}\right) \end{array}$$ (3.1)

and for $\alpha_{2}>\alpha_{1}$,

$$\begin{array}{l} \delta^{D}\left(t,\alpha_{2},\alpha_{1}\right)=N\cdot \left(\alpha_{2}-\alpha_{1}\right)\cdot DE\left(t,\alpha_{1}\right). \end{array}$$ (3.2)

Note $\delta^{D}\left(t,\alpha_{0},\alpha_{1}\right)$ or $\delta^{D}\left(t,\alpha_{2},\alpha_{1}\right)$ is not a meaningful causal estimand by itself because $\text{DE}\left(t,\alpha_{1}\right)$ is conditional on $\alpha_{1}$ only and does not account for change in DE when proportion vaccinated is $\alpha_{0}$ or $\alpha_{2}$ instead of $\alpha_{1}$. Importantly, now direct impact can be a lower bound on overall impact. This is because Theorem 1 decomposes OE into TE, $IE^{Unvax}$, and $IE^{Vax}$, such that we can map direct impact onto $N\cdot \left|\alpha^{\prime }-\alpha \right|\cdot \text{DE}\left(t,\alpha^{\prime }\right)$ in equations (2.1) and (2.2), which are now written as:

$$\begin{array}{l} \begin{array}{ll} & \delta^{O}\left(t,\alpha_{0},\alpha_{1}\right)=N\cdot \alpha_{0}\cdot IE^{Vax}\left(t,\alpha_{0},\alpha_{1}\right)\;\\ & +\delta^{D}\left(t,\alpha_{0},\alpha_{1}\right)+N\cdot \left(1-\alpha_{0}\right)\cdot IE^{Unvax}\left(t,\alpha_{0},\alpha_{1}\right)\; \end{array} \end{array}$$ (4.1)

for $\alpha_{1}>\alpha_{0}$, and

$$\begin{array}{l} \begin{array}{ll} & \delta^{O}\left(t,\alpha_{1},\alpha_{2}\right)=N\cdot \alpha_{2}\cdot IE^{Vax}\left(t,\alpha_{1},\alpha_{2}\right)\;\\ & +\delta^{D}\left(t,\alpha_{2},\alpha_{1}\right)+N\cdot \left(1-\alpha_{2}\right)\cdot IE^{Unvax}\left(t,\alpha_{1},\alpha_{2}\right)\; \end{array} \end{array}$$ (4.2)

for $\alpha_{2}>\alpha_{1}$. Since overall impact has the same sign when $\alpha_{1}$ is compared to a lower ($\alpha_{0}$) or higher ($\alpha_{2}$) hypothetical value, we formalize the assumption often made in the empirical vaccine-averted and -avertible outcome studies—that is, direct impact is a lower bound on overall impact by considering Claim 1:


$$\begin{array}{l} \delta^{O}\left(t,\alpha_{0},\alpha_{1}\right)\geq \delta^{D}\left(t,\alpha_{0},\alpha_{1}\right)\;\;\;\;\;\;\;\forall \;\alpha_{0}<\alpha_{1}, \end{array}$$

and


$$\begin{array}{l} \delta^{O}\left(t,\alpha_{1},\alpha_{2}\right)\geq \delta^{D}\left(t,\alpha_{2},\alpha_{1}\right)\;\;\;\;\;\;\;\forall \;\alpha_{2}>\alpha_{1}. \end{array}$$

If Claim 1 is true, direct impact, which can be estimated using commonly available data,^4–9,18^ is a lower bound on overall impact that is relevant for policymaking and retrospective policy evaluation requiring samples from a population of groups as in two-stage randomized trials.^12^

Following the literature, we consider two special cases of Claim 1. In Claim 1a, a particular vaccination proportion $\alpha_{1}$ is compared to a hypothetical of no vaccination ($\alpha_{0}=0$) to quantify vaccine-averted outcomes.

Claim 1a (vaccine-averted outcomes):


$$\begin{array}{l} \delta^{O}\left(t,0,\alpha_{1}\right)\geq \delta^{D}\left(t,0,\alpha_{1}\right). \end{array}$$

In words, Claim 1a asserts that for $\alpha_{1}>\alpha_{0}=0$, outcomes averted via direct impact of current vaccination among vaccinated individuals is a lower bound on total vaccine-averted outcomes among both vaccinated and unvaccinated individuals.

In Claim 1b, $\alpha_{1}$ is compared to a hypothetical of near-universal vaccination ($\alpha_{2}=0.9>\alpha_{1}$) to quantify vaccine-avertible outcomes, following the literature.^9^

Claim 1b (vaccine-avertible outcomes):


$$\begin{array}{l} \delta^{O}\left(t,\alpha_{1},0.9\right)\geq \delta^{D}\left(t,0.9,\alpha_{1}\right). \end{array}$$

In words, Claim 1b asserts that for $\alpha_{2}=0.9>\alpha_{1}$, outcomes avertible via direct impact of current vaccination among some unvaccinated individuals is a lower bound on vaccine-avertible outcomes among both vaccinated and unvaccinated individuals. Note we do not compare to $\alpha_{2}=1$ because DE would be undefined under full vaccination, and there would not be an epidemic under full vaccination with a highly effective vaccine.

Based on the partitioning in equations (4.1) and (4.2), Claims 1a and 1b hold if $IE^{Vax}$ and $IE^{Unvax}$ are nonnegative. However, it is not immediately intuitive when that occurs. Therefore, we describe a transmission model to check Claims 1a and 1b under various scenarios and then describe conditions under which direct impact is or is not a lower bound on overall impact.

## TRANSMISSION MODEL

### The Susceptible-Infected-Recovered-Death Model With Vaccination at Baseline

A susceptible-infected-recovered-death model represents a well-mixed group in a two-stage randomized trial assuming partial interference.^12^ To simulate direct and overall impact, we simulate a typical group with a large size under a pair of counterfactual vaccination proportions. In this simulation, (marginal) group average potential outcome is equivalent to (marginal) population average potential outcome because there is only one group in the population. The model consists of four states for a vaccinated or unvaccinated individual—susceptible, infectious, recovered, and death due to infection. We assume that the group is randomly assigned to a vaccination policy wherein the proportion to be vaccinated is α, and individuals are randomly assigned a vaccination status a at baseline (a=1 for vaccinated and a=0 for unvaccinated; for equation (5), subscript v denotes vaccinated and u for unvaccinated) based on their group proportion. The vaccine is “leaky” in protecting against infection and infection-related death—that is, vaccination reduces susceptibility by θ against infection (i.e., VE against infection [$VE_{infection}$] is (1−θ)·100%) and reduces susceptibility by κ against death (i.e., VE against death given infection [$VE_{death\;|\;infection}$] is (1−κ)·100%). Individuals mix homogeneously such that each vaccinated or unvaccinated susceptible individual is equally likely to contact any infectious individual. Vaccinated and unvaccinated infectious individuals are equally contagious. The transmission dynamics are:

$$\begin{array}{l} \begin{array}{l} dS_{u,\alpha }\left(t\right)/dt=-\lambda_{\alpha }\left(t\right)\cdot S_{u,\alpha }\left(t\right)\;\\ dS_{v,\alpha }\left(t\right)/dt=-\theta \cdot \lambda_{\alpha }\left(t\right)\cdot S_{v,\alpha }\left(t\right)\;\\ dI_{u,\alpha }\left(t\right)/dt=\lambda_{\alpha }\left(t\right)\cdot S_{u,\alpha }\left(t\right)-\gamma \cdot I_{u,\alpha }\left(t\right)\;\\ dI_{v,\alpha }\left(t\right)/dt=\theta \cdot \lambda_{\alpha }\left(t\right)\cdot S_{v,\alpha }\left(t\right)-\gamma \cdot I_{v,\alpha }\left(t\right)\;\\ dR_{u,\alpha }\left(t\right)/dt=\left(1-\mu \right)\cdot \gamma \cdot I_{u,\alpha }\left(t\right)\;\\ dR_{v,\alpha }\left(t\right)/dt=\left(1-\kappa \cdot \mu \right)\cdot \gamma \cdot I_{v,\alpha }\left(t\right)\;\\ dD_{u,\alpha }\left(t\right)/dt=\mu \cdot \gamma \cdot I_{u,\alpha }\left(t\right)\;\\ dD_{v,\alpha }\left(t\right)/dt=\kappa \cdot \mu \cdot \gamma \cdot I_{v,\alpha }\left(t\right)\; \end{array}\} \end{array}$$ (5)

where γ= recovery rate, $\lambda_{\alpha }\left(t\right)=\beta \cdot \frac{I_{u,\alpha }\left(t\right)+I_{v,\alpha }\left(t\right)}{N\left(t\right)}$ is the hazard rate of infection, with β the number of effective contacts made by a typical infectious individual per unit time, and μ=  probability of death due to infection. In equation (5), $S_{u,\alpha }\left(t\right)$ and $S_{v,\alpha }\left(t\right)$ denote, respectively, the number of susceptible individuals who are unvaccinated and vaccinated, $I_{u,\alpha }\left(t\right)$ and $I_{v,\alpha }\left(t\right)$ for the infectious individuals, $R_{u,\alpha }\left(t\right)$ and $R_{v,\alpha }\left(t\right)$ for the recovered individuals who are no longer at risk, and $D_{u,\alpha }\left(t\right)$ and $D_{v,\alpha }\left(t\right)$ for those who died due to infection. N(t) denotes the sum of all compartments at time t. eFigure 1; http://links.lww.com/EDE/C222 shows the model flowchart, and eTable 1; http://links.lww.com/EDE/C222 shows parameter values used in simulation.

### Software

All simulations and visualization are conducted using R 4.2.2 (R Foundation for Statistical Computing, Vienna, Austria).^22^ All models are implemented using R package *odin*.^23^ Code is available at https://github.com/katjia/impact_estimands.

## WHEN IS DIRECT IMPACT A LOWER BOUND ON OVERALL IMPACT?

### Rationale

The susceptible-infected-recovered-death model has many simplifying assumptions compared to real-world settings. However, if one can show a counterexample to Claim 1 based on the simplest susceptible-infected-recovered-death model, then Claim 1 is not guaranteed to be true in more general and realistic models. To identify counterexamples of Claim 1, we consider common violations to assumptions on time-invariant parameters so that IEs can be negative: (1) number of effective contacts may increase over time due to meteorological factors,^24^ lifting of nonpharmaceutical interventions,^25^ and seasonal variation in social contacts, (2) infection-fatality risk may increase over time,^26^ (3) waning immunity clearly occurred in the Delta and Omicron waves of COVID-19 pandemic.^27,28^

### Scenarios

We check the claims under five scenarios (Table). Scenario 1 refers to the susceptible-infected-recovered-death model in equation (5) with time-invariant parameters. eTable 1; http://links.lww.com/EDE/C222 lists model parameters for which one or two parameters vary under each scenario separately: Scenario 2 increases number of effective contacts made by a typical infectious individual per day (β) from 0.15 to 0.6 from day 300 onwards; Scenario 3 increases probability of death due to infection (μ) from 0.01 to 0.1 from day 300 onwards; Scenario 4 allows both $VE_{infection}$ and $VE_{death\;|\;infection}$ to wane linearly after day 100 reaching 0% at day 300; and Scenario 5 combines Scenarios 2 (increasing β) and 4 (waning VEs).

**TABLE.** Scenarios Under Which the Claims May or May Not Hold^a^

Scenario	Parameters	Claim 1^b^	Claim 1a^b^	Claim 1b^b^
Scenario 1	Time-invariant parameters	+	+	+
Scenario 2	β increases from 0.15 to 0.6 from day 300 onwards	−	−	−
Scenario 3	μ increases from 0.01 to 0.1 from day 300 onwards	−	−	−
Scenario 4	$VE_{infection}$ and $VE_{death\;\;infection}$ wane linearly after day 100, reaching 0% at day 300	−	+	−
Scenario 5	Scenarios 2 and 4 combined	−	−	−

β is the number of effective contacts made by a typical infectious individual per day; μ is the probability of death due to infection; $VE_{infection}$ is the vaccine efficacy against infection; $VE_{death\;\;infection}$ is the vaccine efficacy against death given infection.

a Positive sign (+) indicates that the claim holds; negative sign (−) otherwise.

b Claim 1 holds only if Claims 1a and 1b hold.

### Proof of Claim 1 at the End of Outbreak Under Scenario 1

For Scenario 1 (i.e., time-invariant parameters), in eAppendix 4; http://links.lww.com/EDE/C222, we prove that Claim 1 (i.e., direct impact is a lower bound on overall impact for any two proportions vaccinated) holds in the susceptible-infected-recovered-death model at the end of outbreak (i.e., at t→∞).

### Simulations

For t<∞, we first verify Claims 1a and 1b (special cases of Claim 1) through simulation based on parameters specified in eTable 1; http://links.lww.com/EDE/C222 and initial conditions in eTable 2; http://links.lww.com/EDE/C222. We specify $\alpha_{0}=0$ versus $\alpha_{1}=0.7$ in the pair of trajectories to verify Claim 1a and $\alpha_{1}=0.7$ versus $\alpha_{2}=0.9$ to verify Claim 1b. If Claims 1a and 1b both hold for the specified parameters, Latin hypercube sampling is conducted to generate alternative sets of proportions vaccinated and model parameters to verify the full Claim 1. If only one of Claim 1a or 1b holds, Latin hypercube sampling is conducted to verify whether the Claim holds under alternative proportion vaccinated and model parameters (e.g., trying alternative values for $\alpha_{1}$ while fixing $\alpha_{0}=0$ for Claim 1a).

**FIGURE 2.**
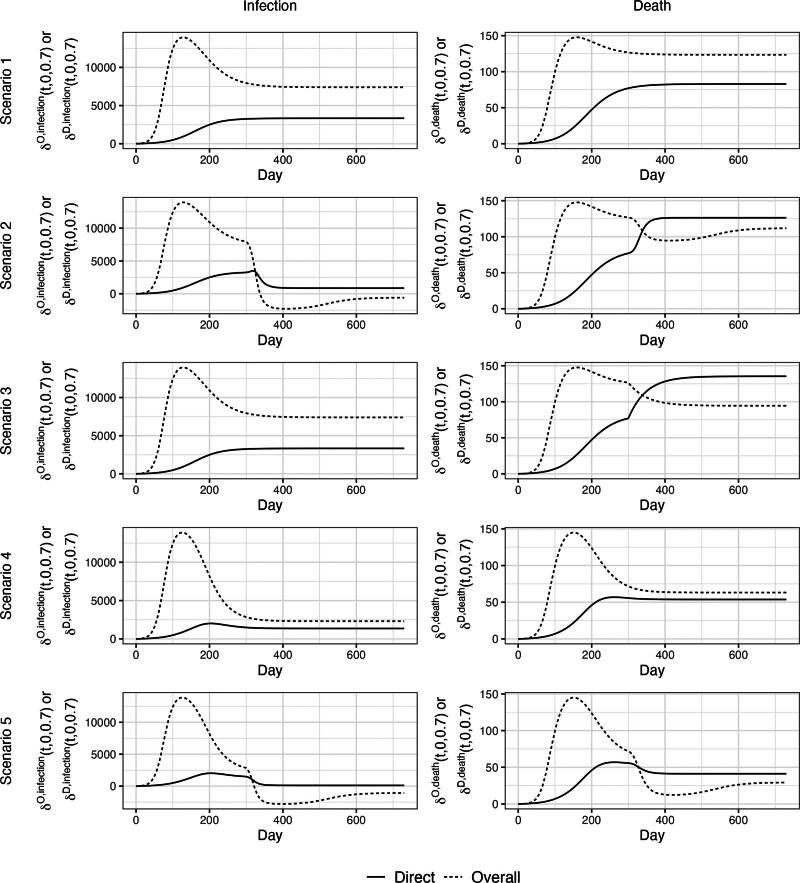
Direct impact and overall impact given $\alpha_{0}=0$ and $\alpha_{1}=0.7$ to verify Claim 1a. In Scenario 1, all parameters are time invariant; in Scenario 2, the number of effective contacts made by a typical infectious individual per day (β) increases from 0.15 to 0.6 at day 300; in Scenario 3, probability of infection-related death (μ) increases from 0.01 to 0.1 at day 300; in Scenario 4, vaccine efficacies against infection and death start to wane linearly after day 100 reaching 0% at day 300; and in Scenario 5, the combination of Scenarios 2 and 4.

**FIGURE 3.**
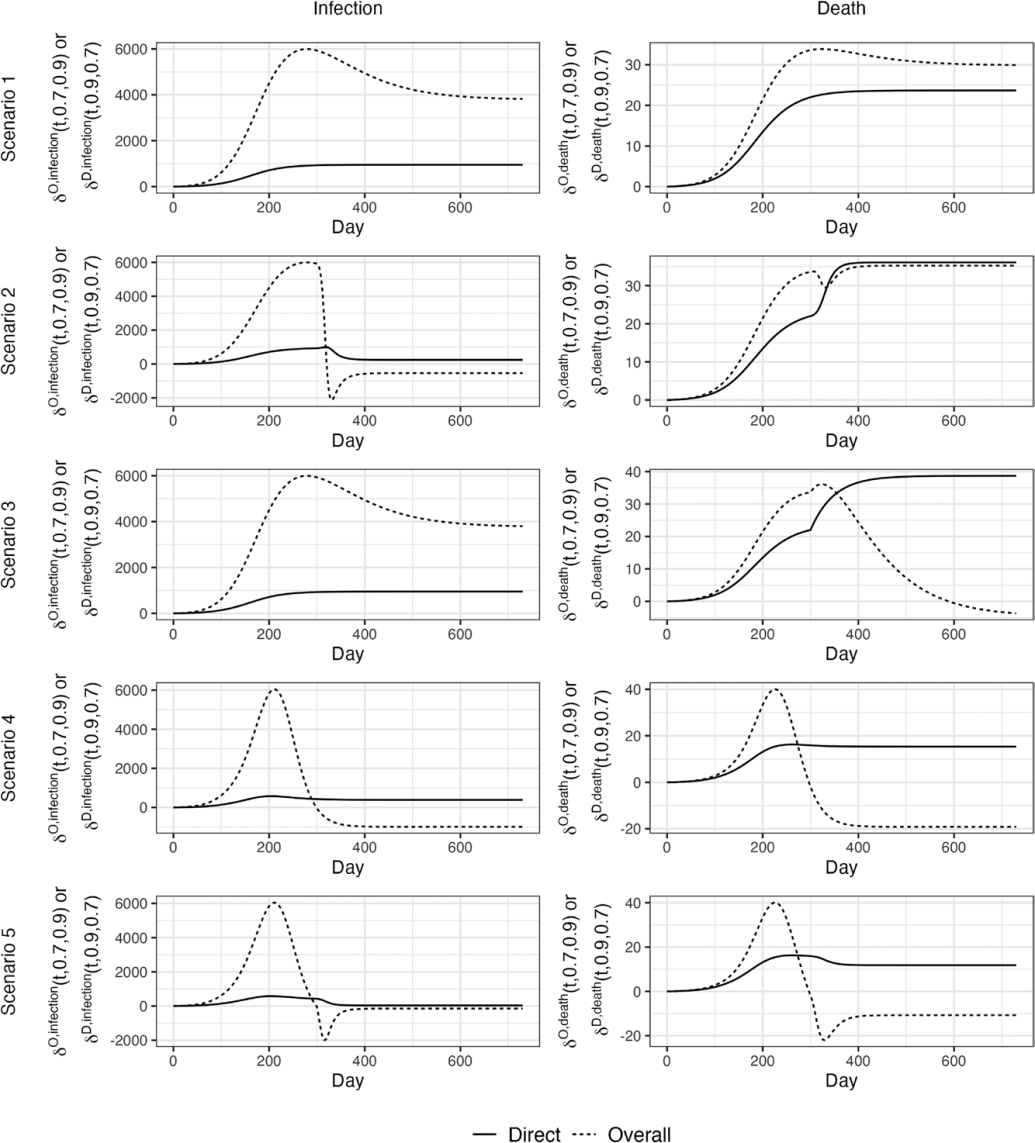
Direct impact and overall impact given $\alpha_{1}=0.7$ and $\alpha_{2}=0.9$ to verify Claim 1b. In Scenario 1, all parameters are time invariant; in Scenario 2, the number of effective contacts made by a typical infectious individual per day (β) increases from 0.15 to 0.6 at day 300; in Scenario 3, probability of infection-related death (μ) increases from 0.01 to 0.1 at day 300; in scenario 4, vaccine efficacies against infection and death start to wane linearly after day 100 reaching 0% at day 300; and in Scenario 5, the combination of Scenarios 2 and 4.

Briefly, Claim 1 only holds under Scenario 1 (i.e., time-invariant parameters), but not under any other Scenarios. Table summarizes the results. Figures 2 and 3 show trajectories of direct and overall impact throughout the epidemic to verify Claims 1a and 1b, respectively. Figures 2 and 3 show that Claims 1a and 1b hold under Scenario 1. Moreover, Latin hypercube sampling verifies that Claim 1 holds under Scenario 1 (eAppendix 5; http://links.lww.com/EDE/C222). Under Scenario 2 where β increases, Claims 1a and 1b do not hold: Direct impacts are not lower bounds on overall impacts (Figures 2 and 3) due to negative IEs (eFigures 3–5; http://links.lww.com/EDE/C222). Under Scenario 3 where μ increases, Claims 1a and 1b hold for infections but not deaths due to negative IEs for death (eFigures 3–5; http://links.lww.com/EDE/C222). Under Scenario 4 where VEs wane, only Claim 1a (vaccine-averted outcomes) holds (Figure 2). Latin hypercube sampling verifies that Claim 1a holds under Scenario 4 (eAppendix 5; http://links.lww.com/EDE/C222). However, Claim 1b (vaccine-avertible outcomes) does not hold (Figure 3) due to negative IEs (eFigures 4 and 5; http://links.lww.com/EDE/C222). Finally, under Scenario 5 where β increases and VEs wane, Claims 1a and 1b do not hold.

## DISCUSSION

Motivated by recent research on deaths averted by COVID-19 vaccination, this study adjusts estimands defined by Hudgens and Halloran to accommodate analyses on averted and avertible outcomes due to infectious disease interventions, thereby enabling researchers to distinguish the estimands when conducting and interpreting related studies. An epidemic model is simulated to verify the commonly held claim that outcomes averted (or avertible) via direct impact among the vaccinated (or unvaccinated) individuals is a lower bound on overall impact. Based on the susceptible-infected-recovered-death model, the lower bound fails when transmission or fatality parameter increases, or VEs wane, implying that the lower bound is not guaranteed to hold for more general and realistic models. Consequently, it would be overly optimistic for empirical studies^4–9^ to assume that they have estimated a lower bound on a true number of averted (or avertible) outcomes through estimating the direct impact without examining the directions of IEs.

When IEs for the vaccinated and unvaccinated are both positive (i.e., higher vaccination coverage yields a positive number of averted or avertible outcomes), direct impact is a lower bound on overall impact (equations 4.1 and 4.2). Subsequently, we show in eAppendix 4; http://links.lww.com/EDE/C222 that, at the end of epidemic, deaths averted via direct impact would be a lower bound on overall impact in a susceptible-infected-recovered-death model given time-invariant parameters and vaccination at baseline, thanks to the positive IEs, a finding proven more recently in a preprint by Lin et al.^29^ In general, IEs may be positive because vaccination reduces the infectious individuals at a given time, such that infection-naive individuals are less likely to be infected.^30^ However, as shown above, there are scenarios under which direct impact is not a lower bound on overall impact due to negative IEs (Scenarios 2–5).

First, when the number of effective contacts made by a typical infectious individual per day (β) increases over time (Scenario 2), the overall impact on infection decreases (and can be negative) because at the early stage of outbreak, the less-vaccinated group has many infected and recovered with sterilizing immunity; while the more-vaccinated group has more susceptible (i.e., infection-naive) individuals who have escaped earlier infections and will experience a higher force of infection at a later time (eFigure 6; http://links.lww.com/EDE/C222). β is affected by meteorological factors,^24^ behavioral factors (e.g., usage of personal protective equipment),^31^ biological factors (e.g., changes in host immunity, evolution of strains),^32^ nonpharmaceutical interventions, and seasonal variation in social contacts.

Second, when the probability of infection-related death (μ) increases over time (Scenario 3), the overall impact on death decreases because the extensively vaccinated group(s) has more who escape earlier infections and then experience higher fatality at a later stage of outbreak (eFigure 6; http://links.lww.com/EDE/C222). It is plausible for the lethality of pathogens to increase over time: Disease severity increased in the autumn wave of the 1918 flu pandemic compared with the spring-summer wave of the same pathogen in that year.^26^ Increasing lethality implies that vaccination at beginning of outbreak can decrease overall impact by postponing cases. On the other hand, if fatality rate increases with infection peak due to the sudden shortage of healthcare resources, overall impact on death will be more positive because vaccines delay infection and flatten the epidemic curve. Likewise, if infection-fatality rates decline progressively due to improvements in care,^33,34^ then vaccination that delays the epidemic can have amplified positive overall impact.

Third, overall impact may become negative when VEs wane (Scenario 4). In particular, vaccination proportion of $\alpha_{2}=0.9$ may result in more infections and deaths than the proportion of $\alpha_{1}=0.7$. eAppendix 8; http://links.lww.com/EDE/C222 discusses this scenario in greater detail.

In addition, if there were multiple risk groups with heterogeneous susceptibility to adverse outcomes and heterogeneous mixing patterns, vaccination for a subgroup could cause negative IE in other subgroups by increasing risk for more severe complications. For example, empirical evidence showed that low rubella vaccination coverage in children increased rubella incidence in the 15-and-over and incidence of congenital rubella in newborns.^35^

The current study has some limitations. First, vaccination is assumed to be a one-time event at baseline before start of outbreak, but in reality, vaccine rollout is continuous over time and may occur during outbreak. Second, throughout, we have considered the case where vaccination occurs at random. However, in most empirical settings there may be strong confounding due to staged rollout of vaccines and differences in vaccine acceptance by behavioral and health characteristics. Such confounding if uncontrolled threatens the validity of inferences about the effects, whether or not a bound is valid in ideal (unconfounded) circumstances.

Another limitation is that in the scenarios considered, changes in lethality and transmission were assumed to occur at a fixed time, whereas in reality, they might well occur either in response to pathogen evolution^36^ or to behavioral changes that are affected by the epidemic trajectory. However, our goal was not to describe the details of a particular epidemic but to describe qualitatively conditions under which infectious disease interventions may not prevent more cases overall than directly. Finally, the susceptible-infected-recovered-death model does not consider deaths due to other factors, meaning that simulations are applicable to studies whose outcome of interest is a consequence of infection. Over a short time frame (e.g., 1 year), it is acceptable to consider deaths due to infection only, when other causes of death are negligible. We also did not consider possible adverse events after vaccination, although adverse events have important policy implications. Our focus here is impact of interventions on averting disease outcomes. Future studies can extend the estimands to investigate adverse events.

In conclusion, this study examines the commonly held assumption in empirical vaccine-averted and -avertible analyses that direct impact is a lower bound on overall impact due to indirect protection. We show that the use of direct impact as a lower bound on overall impact is reliable only under very strong and often unrealistic assumptions. Therefore, it is inadvisable for empirical studies to assume this relation without examining the directions of IEs. Alternatively, if researchers want to estimate the averted and avertible outcomes, a transmission model should be used to capture the overall impact, thereby improving clarity about what is estimated despite the expense of making additional modeling assumptions.

## ACKNOWLEDGMENTS

We acknowledge Dr Michael Hudgens for discussing the study with us and offering insights on the technical details of this manuscript. We also thank Kathy L. Brenner from Harvard T.H. Chan School of Public Health for providing support in writing the manuscript.
